# Supplementary material for: In Silico and In Vitro Analyses of Strawberry-Derived Extracts in Relation to Key Compounds’ Metabolic and Anti-Tumor Effects
Source: Int J Mol Sci. 2025 Apr 8;26(8):3492. doi: 10.3390/ijms26083492 (PMC12026510; doi:10.3390/ijms26083492)
Supplement: Supplementary file 1 [file ijms-26-03492-s001.zip › ijms-3520264-supplementary.pdf]

Article

In silico and in vitro analyses of strawberry derived extracts in relation to key compounds' metabolic and anti-tumor effects

Lucia Camelia Pirvu [1,*], Amalia Stefaniu [1], Sultana Nita [2], Nicoleta Radu [3,4] and Georgeta Neagu [5,*]

[1] National Institute for Chemical Pharmaceutical Research and Development, ICCF, Department of Pharmaceutical Biotechnologies, 112 Vitan, 031299 Bucharest, Romania; lucia.pirvu1@gmail.com (L.C.P.); astefaniu@gmail.com (A.S.)
[2] National Institute for Chemical Pharmaceutical Research and Development, ICCF, Department of Physical- Chemical Analysis and Quality Control, 112 Vitan, Bucharest, Romania; sultananita@ncpri.ro (S.N.)
[3] Biotechnology Faculty, University of Agronomic Sciences and Veterinary Medicine of Bucharest, 59 Marasti, District 1, Bucharest, Romania; nicoleta.radu@biotehnologii.usamv.ro (N.R.)
[4] National Institute of Chemistry and Petrochemistry Research and Development, Department of Biotechnology, 202 Splaiul Independentei, Bucharest, Romania; nicoleta.radu@biotehnologii.usamv.ro (N.R.)
[5] National Institute for Chemical Pharmaceutical Research and Development, ICCF, Department of Pharmacology, 112 Vitan, 031299 Bucharest, Romania; georgetaneagu2008@gmail.com (G.N.)
* Correspondence: lucia.pirvu1@gmail.com (L.C.P.); georgetaneagu2008@gmail.com (G.N.)

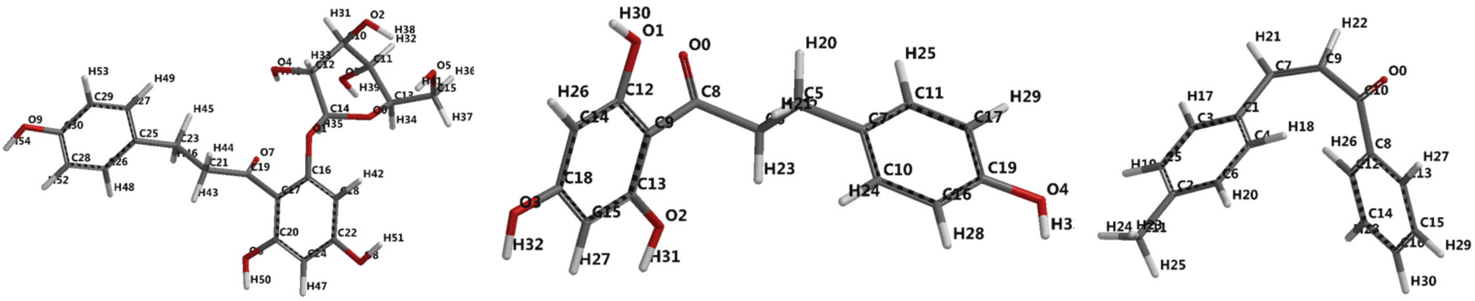

a b c

Figure S1. Labeling minimized structures of phloridzin (a); phloretin (b), and 4-methylchalcone (c).

Citation: To be added by editorial staff during production.

Academic Editor: Firstname Lastname

Received: date
Revised: date
Accepted: date
Published: date

Copyright: © 2024 by the authors. Submitted for possible open access publication under the terms and conditions of the Creative Commons Attribution (CC BY) license (https://creativecommons.org/licenses/by/4.0/).
